# Frequency and severity of autonomic dysfunction assessed by objective hemodynamic responses and patient-reported symptoms in individuals with myasthenia gravis

**DOI:** 10.3389/fnins.2024.1415615

**Published:** 2024-07-19

**Authors:** Monika Zawadka-Kunikowska, Mirosława Cieślicka, Jacek J. Klawe, Małgorzata Tafil-Klawe, Wojciech Kaźmierczak, Łukasz Rzepiński

**Affiliations:** ^1^Department of Human Physiology, Ludwik Rydygier Collegium Medicum in Bydgoszcz, Nicolaus Copernicus University, Bydgoszcz, Poland; ^2^Department of Hygiene, Epidemiology, Ergonomy and Postgraduate Education, Ludwik Rydygier Collegium Medicum in Bydgoszcz, Nicolaus Copernicus University in Torun, Bydgoszcz, Poland; ^3^Faculty of Medical Sciences, Bydgoszcz University of Science and Technology, Bydgoszcz, Poland; ^4^Institute of Physiology and Pathology of Hearing, Warsaw, Poland; ^5^Sanitas - Neurology Outpatient Clinic, Bydgoszcz, Poland; ^6^Department of Neurology, 10th Military Research Hospital and Polyclinic, Bydgoszcz, Poland

**Keywords:** myasthenia gravis, autonomic dysfunction, COMPASS-31 scale, orthostatic, cardiac, composite autonomic scoring scale

## Abstract

**Introduction:**

Myasthenia gravis (MG), a rare autoimmune disorder, poses diagnostic and management challenges, with increasing incidence in Europe and significant impact on patient quality of life. Despite prevalent autonomic symptoms, comprehensive assessments integrating subjective and objective measures are lacking. We aimed to investigate the prevalence and severity of autonomic dysfunction in patients with MG and healthy controls (HCs).

**Materials and methods:**

We used beat-to-beat hemodynamic responses during standardized autonomic function tests (AFTs) and the Composite Autonomic Symptom Score 31 (COMPASS-31) questionnaire. Study participants including, 53 patients with MG and 30 age- and sex matched HCs underwent standardized cardiovascular AFTs and completed the COMPASS-31 questionnaire. Patients were categorized into Non-CAN and CAN groups based on their Cardiovascular Autonomic Neuropathy (CAN) status, as evaluated using the Composite Autonomic Scoring Scale (CASS). During the AFTs, cardiovascular parameters including heart rate, systolic blood pressure (BP), diastolic BP, mean BP, stroke volume (SV), cardiac output (CO), and total peripheral resistance (TPR) were measured.

**Results:**

Twenty patients with MG (38%) exhibited mild CAN (CASS ≥2) with a median total CASS score of 1.00 and CASS 0.00 in HCs. Adrenergic impairment was observed in 27 patients (52%), with 13 patients (24.5%) exhibiting longer pressure recovery time after Valsalva maneuver (VM). Cardiovagal impairment was evident in 71% of patients, with abnormal results observed in 39.6% for the deep breathing test and 56.6% for the VM. CAN MG showed worse scores than HCs for the total COMPASS-31 (*p* < 0.001), orthostatic (OI) (*p* < 0.001), secretomotor (*p* = 0.004), and pupillomotor domains (*p* = 0.004). Total COMPASS-31 and OI scores were correlated with worse disease outcomes (disease duration, severity), hemodynamic parameter changes (SV, CO, TPR) during phase II late of VM, and with changes (Δtilt-supine) in Δsystolic BP, Δdiastolic BP, Δmean BP, ΔTPR during head-up-tilt test, but not with CASS score.

**Conclusion:**

Our findings demonstrate mild cardiovascular autonomic impairment in adrenergic and cardiovagal domains in patients with MG. Additionally, patient-reported autonomic symptoms correlated with hemodynamic changes during AFTs and worse disease outcomes and not with the grade of autonomic abnormalities. Incorporating beat-to-beat hemodynamics during AFTs may offer further insights for characterizing orthostatic intolerance symptoms in MG group.

## 1 Introduction

Myasthenia gravis (MG) is a rare, chronic autoimmune disorder that primarily affects the postsynaptic membrane at the neuromuscular junction, leading to skeletal muscle involvement (Gilhus et al., 2019; Dresser et al., 2021). In Europe, the estimated incidence of MG is increasing, ranging from 0.63 to 2.9 per 100,000 individuals, with a high female-to-male ratio (Sobieszczuk et al., 2021; Mevius et al., 2023). Although MG is associated with an overall increase in life expectancy, patients with MG experience a lower health-related quality of life than the general population (McCallion et al., 2024). In approximately 80% of MG cases, clinical manifestations, including ocular symptoms (ptosis and diplopia), generalized muscle weakness, fatigue, and respiratory insufficiency, are attributed to the presence of muscle nicotinic acetylcholine receptor (nAChR) antibodies. Approximately 10–15% of the patients with MG have antibodies to other neuromuscular junction (NMJ) molecules, whereas 5–10% are classified as seronegative (Gilhus et al., 2019). The chronic nature of MG leads to extra-muscular manifestations, including those regulated by the autonomic nervous system (ANS) (Gilhus et al., 2021). The mechanisms of ANS dysregulation in patients with MG involves complex interactions including systemic inflammation (Uzawa et al., 2016; Wu et al., 2020), autoimmune processes targeting neuronal nAChRs (Balestra et al., 2000; Vernino et al., 2001), respiratory factors (Zawadka-Kunikowska et al., 2023a), and medication side effects (Gilhus et al., 2021). Although autonomic symptoms are not typical manifestations of MG, more than 50% of patients often report orthostatic intolerance (OI), gastrointestinal (GI) problems, and pupillomotor issues (Tsiptsios et al., 2008; Nikolić et al., 2014; Benjamin et al., 2018; Rzepiński et al., 2021). Several studies have described alterations in pupillary responses in subsets of patients with MG (Tsiptsios et al., 2008; Benjamin et al., 2018). However, most studies have focused primarily on cardiovascular autonomic dysfunction. A recent meta-analysis of more than 300 patients with MG showed decreased cardiovascular parasympathetic function, and higher sympathovagal balance, supporting cardiac autonomic dysfunction (Zawadka-Kunikowska et al., 2023b).

Since the Mayo Clinic research group initially described the Composite Autonomic Symptom Scale 31 (COMPASS-31) (Sletten et al., 2012), this self-administered questionnaire has become a common tool for assessing autonomic symptoms in various neurodegenerative and autoimmune-mediated diseases (Hilz et al., 2022), including multiple sclerosis (Drulović et al., 2017), Parkinson’s disease (Ahn et al., 2021), small-fiber polyneuropathy (Treister et al., 2015), and diabetic neuropathy (D’Amato et al., 2020). Autonomic questionnaires are gaining attention from patients’ perspectives; however, they have an uncertain relationship with objective laboratory findings related to the ANS (Low et al., 2004; Novak et al., 2024). For laboratory quantification, the Ewing battery and the Composite Autonomic Scoring Scale (CASS) (Cheshire et al., 2021), developed by Low (2003) are widely used as standard tests for assessing autonomic function (Cheshire et al., 2021). Cardiac autonomic dysfunction has been identified in patients with MG, often with limited use of Valsalva maneuvers (VMs) which reflect cardiovagal and adrenergic function, or a lack of autonomic evaluations using the CASS scoring (Zawadka-Kunikowska et al., 2023b). There are conflicting findings regarding the relationship between autonomic questionnaires and autonomic function tests (AFTs) or CASS. Studies have shown a lack of or weak correlations in diabetic and mixed neurological patient groups (Low et al., 2004; D’Ippolito et al., 2024; Novak et al., 2024). Currently, there is a scarcity of comprehensive assessments of the prevalence and severity of autonomic dysfunction in MG, including subjective and objective measures as well as investigating its relationship with disease outcomes. The simultaneous evaluation of hemodynamic responses, including cardiac and peripheral vascular parameters, during autonomic challenges enhances understanding of patient-reported symptoms and provides informative insights (Cheshire and Goldstein, 2019). Furthermore, assessing the degree of autonomic dysfunction in MG patients will be crucial for clinicians in risk stratification and health management. Therefore, in this study we aimed to investigate the prevalence and severity of autonomic dysfunction in patients with MG and healthy controls (HCs) using beat-to-beat hemodynamic responses during standardized AFTs and the COMPASS-31 questionnaire.

## 2 Materials and methods

### 2.1 Participants

**Figure 1:**
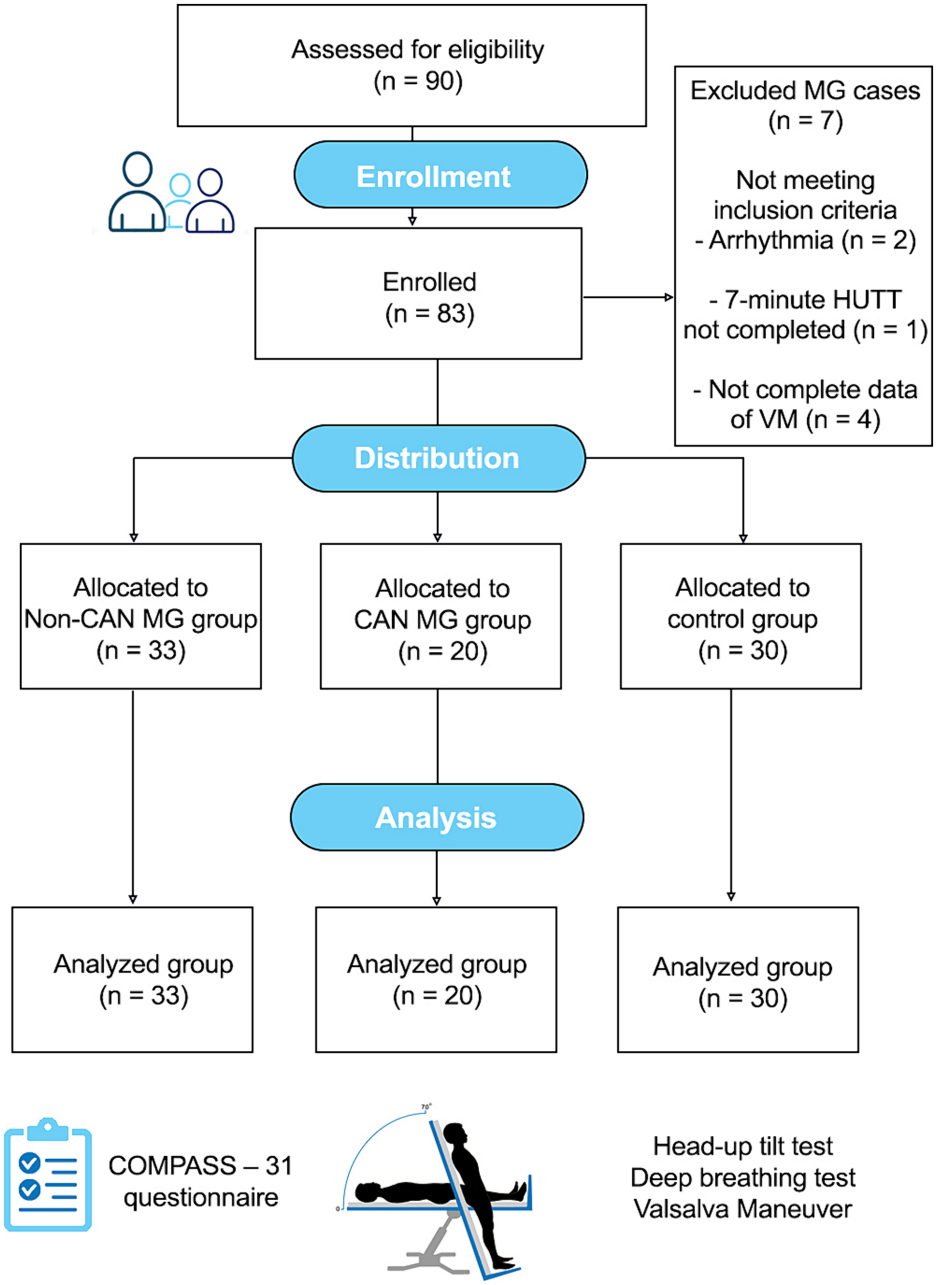
CONSORT flow diagram of the observational study.

We conducted a case–control study of patients with MG attending an outpatient clinic (Sanitas, Bydgoszcz, Poland). The CONSORT flowchart for this study is depicted in Figure 1. Only participants who provided informed consent and met inclusion criteria were included. This study was approved by the Institutional Ethical Committee of the Collegium Medicum at Bydgoszcz, Nicolaus Copernicus University in Torun (KB 747/2017). Demographic characteristics, detailed medical history, signs and symptoms, and laboratory test results of all enrolled patients were obtained from their medical records between 2017 and 2024 (Table 1). The diagnosis of MG was confirmed by neurologists with expertise in neuroinflammatory disorders based on clinical presentation (fatigable limb, bulbar, respiratory, or ocular weakness), abnormal electrophysiology (repetitive nerve stimulation/single-fiber electromyography), positive AChR or MuSK autoantibodies, and clinical response to cholinesterase inhibitors. The inclusion criteria for MG were a diagnosis of MG, no exacerbation of symptoms at the time of assessment, ≥18 years old, no medical history of other disabling pathologies, no other neurological diseases, an absence of previous psychiatric disorders, the ability to estimate self-reported scores independently, and the absence of mechanical ventilation [Myasthenia Gravis Foundation of America (MGFA) classification =5]; worsening of symptoms within the last 30 days was considered a single exacerbation (Rzepiński et al., 2021). The MGFA classification was used to assess the clinical status and severity of MG as follows: pure ocular (class I), mildly generalized (class II), moderately generalized (class III), severely generalized (class IV), and intubation/myasthenic crisis (class V) (Jaretzki et al., 2000). In patients with MG, antibodies against the AChR were detected using an enzyme-linked immunosorbent assay (ELISA). Anti-MuSK IgG4 was identified via ELISA. Thymic pathology was assessed using standard computed tomography imaging and available histological examination of thymic tissue.

**Table 1:** Baseline clinical characteristics of enrolled patients with MG and healthy controls.

	HCs	Total MG	MG	*p*-value*#	HCs
			Non-CAN	CAN	
Number of subjects	30	53	33	20	
Sex, female n (%)	21 (70.0)	45 (84.9)	28 (85)	17 (85)	0.271*
Age, median (Q1–Q3)	38 (25.00–42.00)	41(36.00–45.00)	39 (36.00–45.00)	39.5 (36.00–45.00)	0.481*
Age at onset, median (Q1–Q3)	–	33 (28.00–38.00)	35 (29.00–38.00)	32 (28.00–36.00)	0.126#
Early onset, n (%)	–	36 (67.92)	28 (85.00)	18 (19.00)	0.487#
Disease duration of MG, median (Q1–Q3)	–	3.0 (2.00–8.00)	3 (2.00–7.00)	6.00 (2.509.50)	0.144#
MGFA, median (Q1–Q3)	–	2.0 (1.00–3.00)	2.0 (1.00–2.00)	2.0 (2.00–3.00)	0.033#
Seropositivity to AChR antibodies, n (%)	–	30 (56.6)	16 (48.48)	14 (70%)	0.309#
Seropositivity to MuSK antibodies, n (%)	–	5 (9.43)	3 (9.09)	2 (10.00)	
Double-seropositive, n (%)	–	1 (1.88)	1 (3.03)	0 (0.00)	
Type of MG, n (%)	–				
Ocular	–	8 (15.09)	8 (24.24)	0 (0.00)	
Generalized	–	45 (84.9)	25 (75.75)	20 (100)	
Thymectomy, n (%)	–	17 (32.07)	13 (39.39)	4 (20.00)	
Severity of disease during the testing period (MGFA, %)	–				
Class 0	–	0 (0.00)	0 (0.00)	0 (0.00)	
Class I (ocular)	–	12 (22.64)	11 (33.33)	1 (5.00)	
Class IIa	–	24 (45.28)	13 (39.39)	11 (55.00)	
Class IIIa	–	17 (32.07)	9 (27.27)	8 (40.00)	
Histology changes, n (%)	–				
Thymic pathology	–	33 (62.26)	21 (64)	12 (63)	
Thymoma	–	2 (3.77)	2 (6.25)	0 (0%)	
Unknown	–	2 (3.77)	2 (6.25)	0 (0%)	

MG, myasthenia gravis; MGFA, Myasthenia Gravis Foundation of America classification; CAN, presence of cardiovascular autonomic neuropathy; Non-CAN MG, absence of cardiovascular autonomic neuropathy. Data are presented as medians (Q1, first quartile; Q3, third quartile) or percentages (%). #*p* - value (comparison between CAN vs Non-CAN MG). *p - value (comparison between healthy controls vs. CAN MG and the Non-CAN MG group). AChR, acetylocholine receptor; MuSK, muscle-specific tyrosine kinase.

Exclusion criteria for all the participants were as follows: severe MG stage (MG staging ≥5 and an inability to walk independently), history of cardiovascular events (stroke, ischemic heart disease) diabetes mellitus or drug/alcohol abuse, and treatment with beta-blockers. All participants underwent cardiovascular ANS testing. For ethical reasons, the drugs for MG were continued (Table 1). HCs were primarily recruited from volunteers in the local community of Bydgoszcz, northern Poland. They did not receive any long-term drug therapy and showed no evidence of central or peripheral nervous system diseases or substantial medical history.

### 2.2 COMPASS-31 questionnaire

The COMPASS-31 questionnaire encompasses six different autonomic domains and consists of 100 scores, with a total score ranging from 0 to 100, including 40 pertaining to OI, 5 to vasomotor dysfunction, 15 to secretomotor dysfunction, 25 to GI dysfunction, 10 to urinary dysfunction, and 5 to pupillomotor dysfunction. The weighted sub-scores range from 0 to 5, with a higher score indicating greater autonomic dysfunction (Sletten et al., 2012).

### 2.3 Cardiovascular autonomic function tests

All tests were conducted in our laboratory following recommended methodology and meeting ANS testing criteria (Cheshire and Goldstein, 2019). The tests were conducted in a dark, quiet room at a temperature of 22 ± 1°C, between 8:00 a.m. and 12:00 p.m. No coffee, smoking, alcohol, or exercise was permitted on the test day. All the participants underwent standardized cardiovascular autonomic function testing, assessing cardiovagal and adrenergic functions. Cardiovagal function was evaluated by examining heart rate responses to deep breathing (HRDB) and VMs (Valsalva ratio [VR]) described by Low (2003). Cardiovascular adrenergic function was assessed by evaluating beat-to-beat blood pressure (BP) responses to the VM (phases II and IV) and the presence of orthostatic hypotension (OH) (Low, 1993). OH was defined as a systolic BP (sBP) reduction >20 mmHg or diastolic BP (dBP) reduction >10 mmHg beyond 7 min and 20 s using the passive head-up tilt test (HUTT) at 70°. Postural orthostatic tachycardia syndrome (POTS) is defined as symptoms of OI and exaggerated postural tachycardia (>30 or > 120 bpm) during the HUTT (Thijs et al., 2021). The pressure recovery time (PRT) was calculated as the adrenergic component of baroreflex, representing the time (in seconds) for the systolic BP to recover from phase III back to baseline (Cheshire and Goldstein, 2019). All cardiovascular recordings were obtained from the noninvasive beat-to-beat Task Force Monitor System (CNSystems, Medizintechnik, Graz, Austria), both at baseline in the supine position for 10 min and during AFTs (Fortin et al., 2001). The severity of autonomic impairment was assessed using the cardiovagal and adrenergic sub-scores of Low’s original CASS. In our study, the CASS employed a 0–7 point scale, assigning four points to the adrenergic domain and three to the cardiovagal domain, categorizing autonomic impairments as mild (score ≤ 0), moderate (4–6), and severe (>7). Cardiovascular autonomic neuropathy was characterized by a score of ≥1 in at least two of the modified CASS domains (cardiovagal or adrenergic) or a minimum score of ≥2 in one domain. An abnormal VR, HRDB, was determined using established age-dependent normative ranges for age and sex (Hilz, 2002; Low, 2003). The heart rate (HR) was determined using the ECG signal. Continuous plethysmography was used to record sBP, dBP, and mean BP (mBP). Stroke volume (SV) and cardiac output (CO = SV × HR) were measured using impedance cardiography. The total peripheral vascular resistance (TPR) was determined using the following formula: [(mBP − central venous pressure)/CO] × 80. We calculated HR, sBP, dBP, mBP, TPR, SV, and CO at rest and during VM, as previously described by Hockin et al. (2021a,b): mBP_2A-1_ (magnitude of phase 2A mBP dip relative to phase 1 peak, mmHg), ΔmBP_2B-2A_ (magnitude of phase 2B mBP rise relative to 2A minimum, mmHg), ΔHR_2B-baseline_ (magnitude of phase 2B HR rise relative to baseline, bpm), ΔmBP_4-baseline_ (magnitude of phase 4 mBP overshoot relative to baseline, mmHg), ΔHR_4-baseline_ (magnitude of phase 4 HR decline relative to baseline, bpm), ΔCO_2B-baseline_, ΔSV_2B-baseline_ (magnitude of phase 2B fall in CO and SV relative to baseline, %), and ΔTPR_2B-baseline_ (magnitude of phase 2B TPR increase relative to baseline, %). During HUTT, cardiovascular parameters were recorded at intervals of 3 min 20 s, 5 min 20 s, and 7 min 20 s, with changes (Δtilt-supine) calculated. Individuals with a flattop VM were excluded from the analysis.

### 2.4 Statistical analyses

All statistical analyses were performed using Statistica version 13.3 software. Data are expressed as the median and interquartile range (IQR). Categorical variables are presented as absolute (n) and relative (%) frequencies. Differences in the distribution of categorical variables were determined using the chi-square test or Fisher’s exact test, whereas differences in continuous variables were determined using the nonparametric Mann–Whitney test. The relationships among the CASS, COMPASS-31, and different clinical and demographic factors were evaluated using Spearman’s correlation test. Patients were stratified into two groups according to the absence or presence of CAN (Non-CAN vs. CAN). Multiple comparisons among the CAN MG, Non-CAN MG, and HCs were performed using parametric analysis of variance, followed by the Bonferroni *post-hoc* test, or alternatively, the non-parametric Kruskal–Wallis rank-sum test. Statistical significance was set at *p* < 0.05.

## 3 Results

Fifty-three clinically stable patients with MG and 30 HCs were recruited in this study (Table 1). No significant differences in sex (*p* = 0.106) or age (*p* = 0.251) were observed among the groups. No statistically significant differences were observed among the three groups regarding sex (*p* = 0.271), age (*p* = 0.481), disease duration or age at disease onset, seropositivity to AChR antibodies (Table 1). Disease severity, as measured using the MGFA score, was significantly greater in patients with CAN-MG than in those without. In all subgroups, sex distribution showed female predominance: 85% of Non-CAN patients, 85% of patients with CAN MG, and 70% of HCs were female. Thymic abnormalities were observed in 62.3% of the patients with MG. Generalized MG comprised 84.9% of cases. Anti-AChR antibody positivity was 56.6% and anti-MuSK antibody positivity was observed in 9.4% of MG cases. Pyridostigmine alone was used in 32.1% (17/53) of patients. A total of 30.2% (16/53) of the patients with MG received pyridostigmine and corticosteroids (mean pyridostigmine dose 240 mg/d; prednisone 30 mg/d), and 9.4% (5/53) of the patients with MG required corticosteroids + immunosuppressive agents. In total, 22.6% (12/53) of patients required pyridostigmine + corticosteroids + immunosuppressive agents (7 received azathioprine with a mean dose of 150 mg/d, and 5 r mycophenolate mofetil with a mean dose of 1,000 mg/d). Demographic characteristics, detailed medical histories, laboratory findings, are presented in Table 1.

### 3.1 Comparison of autonomic symptoms (COMPASS-31) between healthy controls and patients with or without CAN

The median COMPASS-31 in the MG group was 26.9 (12.3–36.8). In Table 2, results of the COMPASS-31 questionnaire indicated that patients with CAN MG reported higher overall COMPASS-31 scores (*p* < 0.001) in the orthostatic (*p* < 0.001), secretomotor (*p* = 0.004), and pupillomotor domains (*p* = 0.004). Significant differences in autonomic symptom frequencies, as indicated by the percentage of positive scores, were observed between the groups, with the orthostatic and secretomotor domains being the most affected, whereas the vasomotor domain was less prevalent in the CAN MG group. In contrast, secretomotor, pupillomotor, and GI symptoms were the most frequent complaints in Non-CAN MG patients and HCs. No significant differences were observed in the severity of autonomic symptoms among the MG subgroups (CAN vs. Non-CAN), with *p* > 0.05. Among the six MG patients positive for MuSK antibodies (five seropositive, one double-seropositive), the median (IQR) COMPASS-31 score was 23.0 (5.9–45.1). The severity of autonomic symptoms by domain was: OI (median 16.0, 0.0–28.0), vasomotor (0.0, 0.0–0.8), GI (3.6, 2.6–6.2), secretomotor (4.3, 0.0–8.6), bladder (2.2, 0.0–2.2), and pupillomotor (0.7, 0.0–3.0). The most frequent symptoms were secretomotor, pupillomotor, and GI (5/6, 83%), followed by OI (4/6, 66%), and vasomotor and bladder (2/6, 33%)

**Table 2:** Comparison of cardiovascular autonomic function tests and autonomic symptoms between MG groups and healthy controls.

Autonomic profile	HCs	Total MG	Non-CAN MG	CAN MG	*p*-value
CASS cardiovagal, median [Q1–Q3]	0.00 (0.00–1.00)†	1.00 (0.00–1.00)	0.00 (0.00–1.00)†	1.00 (1.00–1.00)†	<0.001
CASS adrenergic, median [Q1-Q3]	0.00 (0.00–0.00)†	1.00 (0.00–1.00)	0.00 (0.00–0.00)†	1.00 (1.00–1.00)†	<0.001
CASS total, median [Q1–Q3]	0.00 (0.00–1.00)†	1.00 (0–2)	1.00 (1.00–1.00)†	2.0 (2.00–2.00)†	<0.001
Total CASS score 0, n (%)	18 (60)	7 (13.21)	7 (21.21)	0 (0.00)	
Total CASS score 1, n (%)	12 (40)	26 (49.06)	26 (78.78)	0 (0.00)	
Total CASS score 2, n (%)	0 (0)	18 (33.96)	0 (0.00)	18 (90.00)	
Total CASS score 3, n (%)	0 (0)	2 (3.77)	0 (0.00)	2 (10.00)	
PRT, median [Q1–Q3]	2.00 (1.00–3.00)*	3.0 (2.00–5.00)	3.0 (2.00–4.00)	5.00 (2.50–7.00)*	0.002
Valsalva ratio, median [Q1–Q3]	1.57 (1.40–1.80)†	1.4 (2.40–1.17)	1.47 (1.34–1.7)†	1.17 (1.08–1.43)†	
HRDB, median [Q1–Q3]	15.60 (12.8–19.6)†	12.50 (8.50–16.80)	14.3 (10.3–18.00)†	8.65 (6.50–12.80)†	<0.001
Abnormal HRDB, n(%)	1 (3.3)	21 (39.62)	7 (21.21)	14 (70)	<0.002
Abnormal Valsalva ratio, n(%)	9 (30)	30 (56.60)	13 (39.39)	17 (85)	<0.001
Total COMPASS-31 score, median [Q1–Q3]	4.35 (2.78–21.58)*	26.94 (12.29–36.80)	24.16 (8.08–30.04)	33.12 (22.99–39.82)*	<0.001
Orthostatic (OI), median [Q1–Q3]	0.00 (0.00–12.00)*	16.00 (0.00, 20.00)	0.00 (0.00–20.00)	16.12 (16.00–24.00)*	<0.001
>0 [*n* (%)]	8 (27)	34 (64)	15 (45)	19 (95)	<0.001
Vasomotor, median [Q1–Q3]	0.00 (0.00–0.00)	0.00 (0.00–0.00)	0.00 (0.00–0.00)	0.00 (0.00–0.00)	0.042
>0 [*n* (%)]	0 (0)	10 (19)	5 (15)	5 (25)	0.022
Gastrointestinal, median [Q1–Q3]	1.78 (0.89–4.46)	2.67 (0.00–6.24)	2.67 (0.00–6.20)	3.12 (0.89–7.14)	0.721
>0 [*n* (%)]	24 (80)	43 (81)	26 (79)	17 (85)	0.850
Secretomotor, median [Q1–Q3]	0.00 (0.00–4.28)*	4.28 (0.00–6.42)	4.28 (0.00–6.42)	6.42 (3.21–6.42)*	0.003
> 0 [*n* (%)]	13 (43)	43 (81)	24 (73)	19 (95)	<0.001
Bladder, median [Q1-Q3]	0.00 (0.00–1.10)	0.00 (0.00–2.22)	0.00 (0.00–1.11)	1.11 (0.00–2.22)	0.061
> 0 [*n* (%)]	8 (27)	27 (51)	17 (52)	10 (50)	0.098
Pupillomotor, median [Q1–Q3]	0.99 (0.00–1.99)*	2.66 (1.33–3.33)	2.33 (1.33–2.99)	2.83 (1.66–3.33)*	0.004
> 0 [*n* (%)]	22 (73)	43 (81)	28 (85)	15 (75)	0.497

CASS, Composite Autonomic Scoring Scale; HRDB, responses to deep breathing; MG, myasthenia gravis; PRT, pressure recovery time; CAN MG, presence of cardiovascular autonomic neuropathy; Non-CAN MG, absence of cardiovascular autonomic neuropathy. Data are presented as medians (Q1, first quartile; Q3, third quartile) or percentages (%). *p < 0.05 (*post hoc* comparison between CAN MG and healthy controls). †*p* < 0.05 (*post hoc* comparison between healthy controls with CAN MG and the Non-CAN MG group). HRDB, HR responses to deep breathing.

### 3.2 Comparison of cardiovascular autonomic function tests between healthy controls and patients with or without CAN

Cardiovascular autonomic dysfunction, defined as a minimum CASS score of ≥1 in cardiovagal and adrenergic domains, or a score of ≥2 in at least one domain, was present in 37.7% of patients with MG according to standardized cardiovascular AFTs. The median total CASS was 1.0, consistent with mild cardiovascular autonomic impairment. Adrenergic impairment was observed in 27 patients (50.9%), and prolonged Valsalva pressure recovery time (PRT; >6 s) was identified in 13 (24.5%) patients, with median duration of 3.0 s. Cardiovagal impairment was noted in 66.6% of patients, with a median CASS vagal score of 1. The median total CASS, adrenergic, and cardiovagal scores in HCs were all 0.0 (Table 2).

Patients with CAN MG had higher total CASS, adrenergic, and cardiovagal scores than those in the Non-CAN MG and HCs groups *p* < 0.001 (Table 2). Additionally, 39.6% of MG patients (21/53) exhibited abnormal HRDB, and 56.6% (30/53) had abnormal VR. The CAN-MG group showed significantly lower VR and HRDB values than the Non-CAN MG group (*p* = 0.043 and *p* = 0.003, respectively) and HCs (*p* = 0.005 and *p* < 0.001, respectively). No significant differences in HRDB variation or VR were observed between patient groups, *p* > 0.05.

At baseline before VM, both MG groups (CAN, Non-CAN) had significantly lower values of SV at baseline than HCs (*p* < 0.001; *p* = 0.014), respectively. The CAN MG group showed significantly higher HRs (*p* = 0.003), TPR (*p* = 0.004), dBP (*p* = 0.019), and mBP (*p* = 0.016) than HCs. In contrast, no significant differences were observed between the MG and HC groups in sBP parameters (*p* > 0.05) (Table 3).

**Table 3:** Comparison of cardiovascular hemodynamic parameters between MG groups and healthy controls.

Cardiovascular measures	HCs	Non-CAN MG	CAN MG	*p*-value
Supine HR (bpm)	65.0 (60.0–70.00)*	70.0 (65.0–75.0)	73.0 (66.0–82.0)*	0.005
Supine sBP (mmHg)	108.0 (95.0–122.0)	112.0 (102.00–122.0)	121.0 (114.0–124.0)	0.060
Supine dBP (mmHg)	71.5 (61.0–83)*	76.0 (71.0–84.0)	84.0 (74.5–92.5)*	0.023
Supine mBP, (mmHg)	88.0 (74.0–100.0)*	91.0 (86.0–101.0)	97.5 (90.0–106.5)*	0.033
Supine CO, (l/min)	5.9 (5.1–7.7)	5.5 (4.8–6.3)	5.1 (4.4–6.1)	0.066
Supine SV, (ml)	97.0 (74.0–110.0)†	80.0 (66.0–91.0)†	69.1 (58.0–68.0)†	<0.001
Supine TPR (dyn*s/cm^5^)	1109.0 (865.0–1393.0)*	1303.0 (1021.0–1,520)	14,915 (1211.0–1907.0)*	0.005
ΔmBP_2A phase-1_, (mmHg)	−16.0 (−22.0– −9.0)	−17.0 (−24– −9.0)	−20.5 (−28.5– −11.5)	0.592
ΔmBP_2Bphase-2A_, (mmHg)	19.5 (7.0,23.0)*	11.0 (6.00–21.00)	8.0 (4.5–3.5)*	0.028
ΔmBP_4-baseline_, (mmHg)	18.5 (13.0–21.0)	16.0 (10.0–32.0)	13.0 (7.5–22.0)	0.153
ΔHR_2B-baseline_, (bpm)	27.0 (15.0–35.0)*	25.0 (15.0–33.0)	11.0 (7.0,21.5)*	0.293
ΔHR_4-baseline_, (bpm)	−9.0 (−11.0– −5.0)#	−10.0 (−17.0– −5.0)#	−2.0 (−10.0–2.0)	0.030
ΔCO_2Bphase-2A_ (%)	−14.1 (−26.3– −1.9)	−10.6 (−18.0– −5.7)	−6.7 (−18.3–6.7)	0.329
ΔSV_2Bphase-baseline_, (%)	−31.1 (−43.0– −21.0)*	−28.0 (−38.2– −16.9)	−16.7 (−30– −6.1)*	0.007
ΔTPR_2Bphase-2A_, (%)	1109.0 (865.0–1393.0)*	29.7 (2.2–39.6)	12.5 (−9.3–18.5)*	0.021
**Head-up tilt**				
ΔHR_3min_ (bpm)	15.0 (11.1–20.1)	10.0 (6.2–17.0)	10.0 (7.2–15.1)	0.118
ΔsBP_3min_ (mmHg)	18.33(12.0–25.6)	15.1 (4.9–17.5)	10,(−1.0–18.9)	0.048
ΔdBP_3min_ (mmHg)	25.13 (19.0–34.2)†	17.8 (8.6–26.7)†	18.1 (7.9–23.0)†	<0.001
ΔmBP_3min_ (mmHg)	22.9 (16.8–27.1)†	15.7 (9.4–21.8)†	16,0 (4.4–20.3)†	0.001
ΔTPR_3min_ (%)	42.3 (32.4–58.6)	34.3 (11.3–50.3)	25.6 (7.1–61.2)	0.092
ΔHR_5min_ (bpm)	15.0 (11.1–20.7)	11.0 (6.8–18.9)	12.0 (6.6–16.2)	0.971
ΔsBP_5min_ (mmHg)	16.2 (9.8–12.6)	15.8 (3.2–22.1)	4.1 (−1.0–17.6)	0.142
ΔdBP_5min_ (mmHg)	20.3 (15.4–30.5)†	14.8 (10.3–22.1)†	11.7 (7.1–20.5)†	0.026
ΔmBP_5min_ (mmHg)	17.7 (13.0–25.9)*	15.0 (6.3–19.4)	9.1 (4.5–20.6)*	0.017
ΔTPR_5min_ (%)	36.8 (21.2–60.8)	27.3 (8.2–45.0)	16.1 (5.4–47.2)	0.036
ΔHR_7min_ (bpm)	17.0 (10.9–20.9)	13.0 (7.3–18.4)	12.0 (8.9–17.2)	0.110
ΔsBP_7min_ (mmHg)	14.5 (8.6–19.3)*	10.6 (3.7–19.2)	5.3 (0.1–13.0)*	0.031
ΔdBP_7min_ (mmHg)	18.3 (13.9–22.1)†	12.7 (9.3–15.6)†	10.8 (4.1–18.0)†	0.003
ΔmBP_7min_ (mmHg)	16.2 (12.2,21.0)*	10.6 (8.1–16.1)	8.4 (2.6–16.3)*	0.007
ΔTPR_7min_ (%)	37.8 (18.1–57.6)	23.2 (11.9–42.3)	21.0 (−2.1–43.0)	0.047

Data are presented as medians (Q1, first quartile; Q3, third quartile) or percentages (%). *p < 0.05 (*post hoc* comparison between CAN MG and healthy controls). #*p* < 0.05 (*post hoc* comparison between Non-CAN MG and healthy controls). †*p* < 0.05 (*post ho*c comparison between healthy controls with CAN MG and the Non-CAN MG group). HR, heart rate; sBP, systolic blood pressure; dBP, diastolic blood pressure; mBP, mean blood pressure; SV, stroke volume; CO, cardiac output; MG, myasthenia gravis; TPR, total peripheral vascular resistance; change (Δtilt-supine), mBP_2A-1_, magnitude of phase 2A mBP dip relative to phase 1 peak_;_ ΔmBP_2B-2A_, magnitude of phase 2B mBP rise relative to 2A minimum, mmHg; ΔHR_2B-baseline_, magnitude of phase 2B HR rise relative to baseline; ΔmBP_4-baseline_, magnitude of phase 4 mBP overshoot relative to baseline; ΔHR_4-baseline_, magnitude of phase 4 HR decline relative to baseline; ΔCO_2B-baseline_, magnitude of phase 2B fall in CO relative to baseline; ΔSV_2B-baseline_, magnitude of phase 2B fall in SV relative to baseline; ΔTPR_2B-baseline_, magnitude of phase 2B TPR increase relative to baseline; CAN MG, presence of cardiovascular autonomic neuropathy; Non-CAN MG, absence cardiovascular autonomic neuropathy.

**Figure 2:**
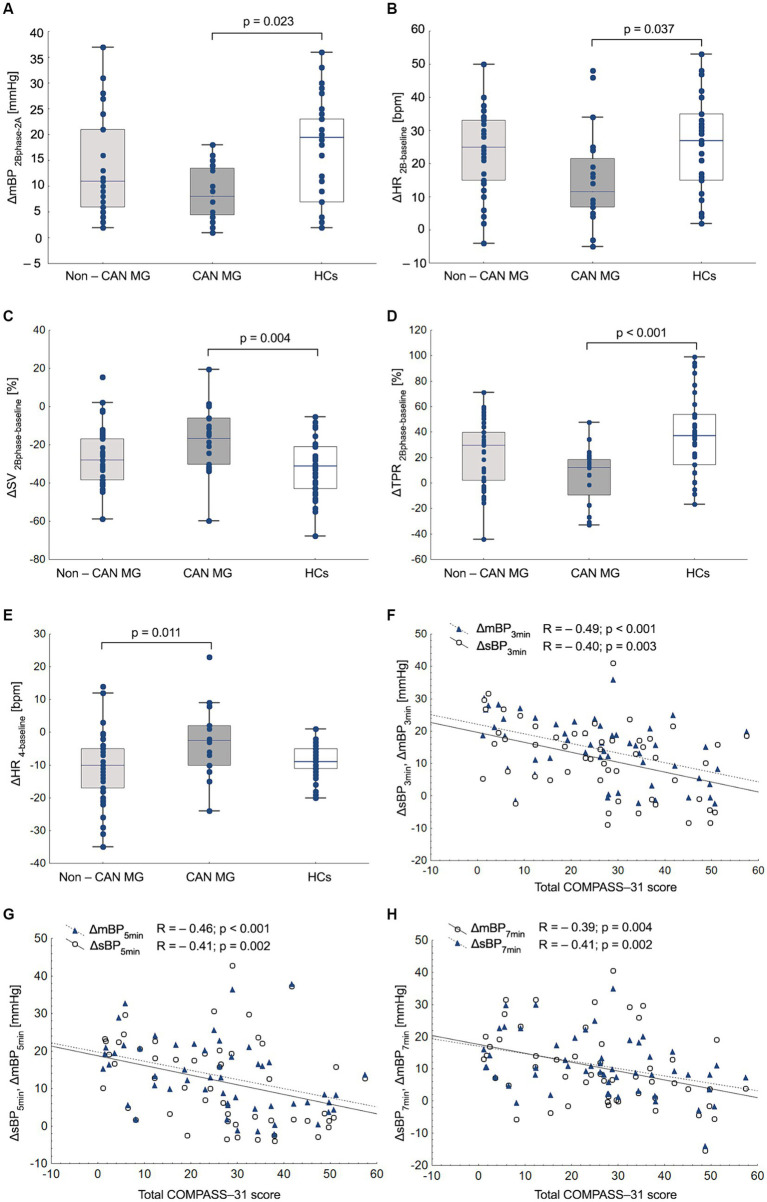
Analysis of hemodynamic responses during the Valsalva maneuver between myasthenia gravis (MG) and healthy controls (HCs). ΔmBP_2B-2A_ (magnitude of phase 2B mean blood pressure rise relative to 2A minimum, mmHg), **(A)**; ΔHR_2B-baseline_ (magnitude of phase 2B heart rate rise relative to baseline, bpm) **(B)**; ΔSV_2B-baseline_ (magnitude of phase 2B fall in stroke volume relative to baseline) **(C)**; ΔTPR_2B-baseline_ (magnitude of phase 2B total peripheral resistance increase relative to baseline), **(D)**; ΔHR_4-baseline_ (magnitude of phase 4 heart rate decline relative to baseline, bpm). **(E)** Relationships between changes in systolic blood pressure (ΔsBP) and mean blood pressure (ΔmBP) during the head-up tilt test (HUTT) with COMPASS-31 score in patients with MG. **(F–H).**

During VM, MG, and HC subgroups exhibited a similar reduction in mBP during phase 2A relative to the phase 1 peak (ΔmBP_2A-1_) and a decrease in CO during phase 2A (ΔCO_2B-2A_). Additionally, no differences were observed between the groups in the increase in mBP during the phase 4 BP overshoot (ΔmBP_4-baseline_). The CAN MG group showed a significantly smaller increase in HR, TPR, and mBP, along with a lower decrease in SV during phase 2 (relative to phase 2A) than HCs (Table 3; Figure 2). Moreover, the decrease in HR during phase 4 bradycardia was significantly lower in the CAN MG group than in Non-CAN MG group (Figure 2). The change in ΔdBP at all phases (3 min: both *p* < 0.001; 5 min: *p* = 0.015, *p* = 0.006; 7 min: *p* = 0.025, *p* = 0.003) of the HUTT and that in ΔmBP at 3 min (*p* = 0.003, *p* = 0.001) were smaller in the CAN and Non-MG groups than in HCs. Specifically, the CAN MG group exhibited a significantly smaller increase in ΔmBP at 5 min (*p* = 0.017) and 7 min (*p* = 0.007), along with lower values of ΔsBP at 7 min (*p* = 0.048), than HCs. Additionally, no differences were observed in HR across all HUTT phases or in ΔsBP at 3 and 5 min after HUTT. Significant differences in the magnitude of change in TPR at 5 and 7 min were observed between groups (*p* > 0.05) (Table 3).

### 3.3 Correlation analysis between clinical and demographical factors and autonomic function indices

In patients with MG, no associations were observed between patient reported autonomic symptoms (COMPASS-31 score, OI sub-score), age, and CASS scores (*p* > 0.05). Orthostatic symptoms were positively correlated with disease duration (*R* = 0.41; *p* = 0.002) and MGFA severity scores (*R* = 0.28; *p* = 0.045). The COMPASS-31 and OI scores were negatively correlated with sBP, dBP, mBP, and TPR at 3, 5, and 7 min during the HUTT (Supplementary Figure S1, Figure 2). Similarly, COMPASS-31 and OI scores were correlated with hemodynamic parameters at rest, including CO (*R* = −0.35; *p* = 0.011, *R* = −0.36; *p* = 0.008), SV (*R* = −0.32; *p* = 0.021, *R* = −0.33; *p* = 0.015), TPR (*R* = 0.27; *p* = 0.048), and during the VM: ΔTPR_2B-baseline_ (*R* = −0.28; *p* = 0.043, *R* = −0.36; *p* < 0.01), ΔSV_2B-2A_ (*R* = 0.41; *p* = 0.002, *R* = 0.34; *p* = 0.012), and ΔCO_2B-2A_ (*R* = −0.28; *R* = 0.32, *p* < 0.05) (Supplementary Table S1).

## 4 Discussion

The 2021 consensus statement from the American Autonomic Society and American Academy of Neurology approved CASS as a valuable scoring system for assessing the severity of autonomic impairment (Cheshire et al., 2021). Currently, limited data exist on CAN prevalence among clinically stable patients with MG, considering the severity of CASS and patient-reported symptoms (e.g., COMPASS-31). Our findings demonstrated that approximately 38% of patients with MG exhibited cardiovascular autonomic impairment (CASS ≥2) with mild severity in the adrenergic and cardiovagal domains. Furthermore, patient-reported autonomic symptoms were correlated with hemodynamic changes during AFTs and worse disease outcomes, although they did not show an association with the CASS.

In the current study, the reported prevalence of parasympathetic dysfunction varied, with 39.6% for deep breathing test (DBT) and 56.6% for the VM. This observation corroborates the findings of earlier cohorts from Serbia (52% for DBT, 44% for Valsalva) (Peric et al., 2011) and Turkey (53.3% for DBT, 0% for Valsalva) (Kocabas et al., 2018). Additionally, Nikolić et al. reported a higher frequency of sympathetic dysfunction than our study (50%), with lower prevalence of parasympathetic dysfunction in AChR-positive patients with MG, with (sympathetic 80.6% vs. parasympathetic 26.9%) and without thymoma (64% vs. 28%), and in MuSK MG cases (78.9% vs. 15.8%) (Nikolić et al., 2014). The variability in autonomic dysfunction can be influenced by respiratory function (Zawadka-Kunikowska et al., 2023a), concurrent MG medications (Gilhus et al., 2021), and mild forms of CAN. Prior studies on MG have primarily focused on sympathetic adrenergic function, typically assessing BP responses to handgrip tests or orthostatic challenges (Zawadka-Kunikowska et al., 2023b), without addressing hemodynamic alterations during VM. The HUTT and VM provide valuable insight into the integrity of the adrenergic component of the baroreflex, inducing a reduction in preload and relative hypotension, thereby stimulating increased sympathetic tone via the arterial and cardiopulmonary baroreflexes (Vogel et al., 2005; Christensen et al., 2022). In our study, reduced BP recovery, blunted compensatory tachycardia, and reflex vasoconstriction during phase II_L, and a longer PRT during the VM, differentiated the CAN MG group from HCs. A similar, although not statistically significant trend in the blunted increase in mBP (mBP_4-baseline_) overshoot during phase IV was also observed between the groups. These indices, indicative of sympathetic integrity, alongside lower frequencies of orthostatic dysautonomia symptoms (POTS and OH), may suggest a mild or early manifestation of adrenergic baroreflex impairment in this subset of patients (La Rovere et al., 2011). Notably, potentially reduced vagal baroreflex responses may be confirmed by attenuated bradycardia in phase 4 in patients with CAN MG compared to those with Non-CAN MG (Hilz et al., 2022). Only one study investigated VM responses in patients with MG. In that study, participants with MG exhibited similar gradual tachycardia (phases II and III), lower BP in phase III, and steep BP overshoot in phase 4 accompanied by bradycardia. The authors postulated the presence of a delayed yet overactive sympathetic activity with normal parasympathetic responses. In contrast to our study, no significant difference in the VR was observed between the patients and controls (Shukla et al., 2013).

Correct adjustment of the CO and TPR levels significantly regulates normal BP in the baseline hemodynamic state and its response to AFTs (Fu et al., 2004; Charkoudian et al., 2005). In our study, data from the HUTT replicated the hemodynamic responses observed during the VM, exhibiting significantly lower BP responses (ΔdBP, ΔmBP) in both MG patient groups than in HCs. A similar trend in lower TPR response increases, albeit not significant, during the HUTT may be explained by preserved vasoconstrictor reserves, potentially contributing to individual variability in OI (Fu et al., 2004). Considering the small vascular responses to VM and HUTT, as well as the decrease in SV during VM (phase II_L) in the CAN group compared to HCs, these findings suggest susceptibility to syncope and/or OI symptoms in the CAN MG group (Hockin et al., 2021a,b). Individuals with CAN exhibited higher resting HR, dBP, mBP, and TPR than healthy controls, indicating lower vagal tone and adrenergic hyperactivity mediated by myocardium (β1) and peripheral vascular smooth muscle adrenergic receptors activation (Sharma and Farrar, 2020). The lower baseline SV in the CAN MG group aligns with findings suggesting a strong inverse relationship between CO and SV, and sympathetically mediated vasoconstriction, as indicated by the higher resting TPR (Charkoudian et al., 2005). The lower SV at rest in both MG groups compared with HCs may result from decreased plasma volume, increased afterload, altered contractility, and potential changes in myocardial geometry (Rowell, 1993). Collectively, our observations align with a recent meta-analysis of eight studies (301 patients with MG and 454 HCs), indicating lower cardiovagal baroreflex sensitivity at rest, diminished parasympathetic (heart rate variability, expiration/inspiration ratio) indices, higher resting sBP, and higher sympathovagal balance in patients with MG at rest and during the HUTT (Zawadka-Kunikowska et al., 2023b). Despite considerable heterogeneity in time-domain heart rate variability measurements and the Expiration/Inspiration ratio among the included studies, the results confirmed our findings of mild alterations in adrenergic and cardiovagal function in the MG group, as revealed by the Valsalva and HUTT indices. It should also be noted that the interpretation of sympathovagal balance via LF/HF has been criticized due to the intricate nature of LF power, its limited correlation with sympathetic nerve activation, and the non-linear interactions between sympathetic and parasympathetic nerve activities (Billman, 2013). Moreover, the Polyvagal Theory, proposed by Porges, elucidates the bidirectional communication between the body’s autonomic responses and the brainstem structures, emphasizing the pivotal role of the vagus nerve in social behavior, emotional regulation, and stress responses (Porges, 2021). It posits that the autonomic state functions as an intervening variable (Porges, 2023) and suggests that reduced heart rate variability is not only an indicator but also a significant homeostatic mechanism in pathological conditions (Ernst, 2017). No correlation was observed between COMPASS-31 and CASS scores (Novak et al., 2024), consistent with a recent large-scale study using subjective and objective assessments of autonomic dysfunction (2,627 patients assessed for autonomic symptoms, CASS, sudomotor tests, skin biopsies, and 564 patients with COMPASS-31). This discrepancy may be attributed to autonomic questionnaires potentially overestimating or underestimating deficits (Novak et al., 2024), because they cover a broader range of symptoms, including GI/bladder issues. Similarly, earlier studies on patients with diabetes showed an overall weak correlation between CASS scores and autonomic symptoms using the Autonomic Symptom Profile questionnaire (Low et al., 2004). In another study (D’Ippolito et al., 2024), COMPASS-31 exhibited a weak association with CAN in type 1 diabetes mellitus and type 2 diabetes mellitus indicating a multifactorial origin of symptoms. In our study, patient-reported autonomic symptoms (total COMPASS-31 score and OI score) were correlated with hemodynamic changes (ΔBP and ΔTPR) induced by AFTs. The strongest association was observed in the change in BP responses during the 3 and 5-min intervals (*R* = −0.39 to −0.5), of the HUTT, suggesting the potential clinical significance of incorporating beat-to-beat hemodynamics in patients reporting OI symptoms (i.e., light-headedness). These observations align with our previous findings suggesting altered BP variability, both at rest and in response to orthostatic stress, which is related to autonomic symptoms and disease severity (Zawadka-Kunikowska et al., 2023b). Subjective OI symptoms showed a stronger correlation with disease duration than its severity (measured by MGFA), possibly involving interference from weakness, fatigue, age, and limited physical activity.

To our knowledge, only a few previously published studies have assessed subjective dysautonomia in patients with MG using the COMPASS-31. However, these MG studies of autonomic dysfunction did not include healthy participants as controls. The total score in our MG population exceeded that reported by Falcão de Campos et al. (mean raw 17.04, weighted ±20.85) (Falcão de Campos et al., 2019) among 24 patients, and by the study conducted by Benjamin et al. (weighted mean 19.5) involving 16 MG patients with myasthenic crisis (Benjamin et al., 2018). Consistent with AFT results, patients with CAN MG exhibited a heightened overall autonomic symptom burden, particularly evident in the orthostatic, secretomotor, and pupillomotor domains compared to HCs. Our observations align with those reported by Benjamin et al. who identified that the most common autonomic dysfunctions were GI (80%), orthostatic dizziness (67.7%), and oculomotor dysfunction (67.7%). The higher frequency of secretory symptoms (increased sweating, dry mouth, dry eyes), and greater severity of pupillomotor symptoms (blurred vision) are partially explained by the side effects of anticholinergic treatment used by patients with MG (Gilhus et al., 2021). In support of Falcão de Campos’ Sudoscan study, no significant differences in foot or hand electrochemical skin conductance (ESC) measurements were observed between patients who discontinued anticholinesterase agents before the sudomotor assessment and controls. Additionally, no significant correlation was observed between the ESC measurements and COMPASS-31. The frequency of vasomotor symptoms differed between groups, although it was the least prevalent domain among all autonomic dysfunction symptoms (Benjamin et al., 2018). The frequency of GI symptoms was similar between the patients and HCs, suggesting that the GI domain may be sensitive to positive responses, even among the healthy population (Foschi et al., 2021). More than 44% of patients with MG use acetylcholinesterase inhibitors and immunomodulating therapies (corticosteroids and azathioprine/mycophenolate mofetil), with abdominal pain, diarrhea, nausea, and flushing being the most common side effects (Sanders and Evoli, 2010). Autonomic function, including decreased baroreflex sensitivity and α1-adrenergic vasoconstrictor response, worsens with age, yet no relationship was found between COMPASS-31 scores and age across all groups. Consequently, patients with MG may exhibit selective impairment in the sympathetic and parasympathetic components of the ANS compared to HCs during the disease, as demonstrated by the COMPASS-31 in our study (Low and Singer, 2023). Dysregulated inflammatory responses in autonomic dysfunction, including chronic inflammation by Th17 cells, B cell maturation and activation by Tfh cells, and Treg cell dysfunction, have been implicated in MG pathogenesis (Uzawa et al., 2021). Nicotinic acetylcholine receptors are crucial for synaptic transmission and are found in neuromuscular junctions, peripheral autonomic ganglia, and pre- and postsynaptic regions in the central nervous system (Vernino et al., 2001). Balestra et al. identified antibodies against α7- and α3-nAChRs in 5 of 60 patients with MG, suggesting a potential association with various clinical features, including autonomic symptoms (Balestra et al., 2000). This implies that pathogenic antibodies against neuronal nAChRs in the MG may be uncommon.

Our study has some limitations. First, the autonomic function assessment was restricted to cardiovascular testing, and sudomotor function was not considered. Second, the COMPASS-31 reflects nonspecific patient-reported feelings, and certain domains might have been overestimated; its use for the quantitative assessment of autonomic symptoms in patients with MG has not been validated. Third, pyridostigmine has vagotonic action, reduces adrenergic sympathetic transduction, and improves the supine cardiovagal baroreflex, increasing systemic resistance, which may mask true ANS deficits. Additionally, the HRDB and VM require sufficient respiratory muscle strength. Fourth, we used a modified CASS with the adrenergic score assessed during the 7-min HUTT. Fifth, the studied HCs and MG population may not be representative of the general population, and the findings may not be generalizable.

## 5 Conclusion

Our results showed that patients with MG exhibited mild cardiovascular autonomic impairment in the adrenergic and cardiovagal domains. Additionally, patient-reported autonomic symptoms were correlated with hemodynamic changes during AFTs and worse disease outcomes, and not with the grade of autonomic abnormalities. This study highlights the utility of CASS scoring in estimating cardiovascular impairment in individuals with MG. Incorporating beat-to-beat hemodynamics, including cardiac and peripheral vascular resistance parameters, during autonomic testing may offer further insights for characterizing orthostatic intolerance symptoms in this group.

## Data availability statement

The original contributions presented in the study are included in the article/Supplementary material, further inquiries can be directed to the corresponding authors.

## Ethics statement

The study was approved by the Bioethics Committee of the Ludwik Rydygier CollegiumMedicumin Bydgoszcz, Nicolaus Copernicus University in Torun, and was performed in accordance with the principles of the 1964 Declaration of Helsinki (747/2017). The studies were conducted in accordance with the local legislation and institutional requirements. The participants provided their written informed consent to participate in this study.

## Author contributions

MZ-K: Conceptualization, Formal analysis, Investigation, Methodology, Project administration, Software, Writing – original draft, Writing – review & editing. MC: Investigation, Writing – review & editing. JK: Supervision, Writing – review & editing. MT-K: Supervision, Writing – review & editing. WK: Supervision, Writing – review & editing. ŁR: Investigation, Methodology, Writing – review & editing.
