# Supplementary material for: Frequency and severity of autonomic dysfunction assessed by objective hemodynamic responses and patient-reported symptoms in individuals with myasthenia gravis
Source: Front Neurosci. 2024 Jul 19;18:1415615. doi: 10.3389/fnins.2024.1415615 (PMC11295281; doi:10.3389/fnins.2024.1415615)
Supplement: Supplementary file 1 [file Table_1.pdf]

Supplementary Material

Supplementary Table 1. Correlation analysis between the COMPASS-31 score and the orthostatic intolerance (OI) sub-score.

	Compass-31		OI score	
	R	p	R	p-value
ΔHR_{3min} (bpm)	-0.04	0.783	0.15	0.300
ΔsBP_{3min} (mmHg)	**-0.40**	**0.003**	**-0.34**	**0.012**
ΔdBP_{3min} (mmHg)	**-0.50**	**0.000**	**-0.40**	**0.003**
ΔmBP_{3min} (mmHg)	**-0.49**	**0.000**	**-0.42**	**0.002**
ΔTPR_{3min} (%)	-0.25	0.076	**-0.27**	**0.049**
ΔHR_{5min} (bpm)	-0.01	0.951	0.23	0.092
ΔsBP_{5min} (mmHg)	**-0.41**	**0.002**	**-0.33**	**0.017**
ΔdBP_{5min} (mmHg)	**-0.49**	**0.000**	**-0.40**	**0.003**
ΔmBP_{5min} (mmHg)	**-0.46**	**0.000**	**-0.38**	**0.005**
ΔTPR_{5min} (%)	**-0.30**	**0.030**	**-0.35**	**0.009**
ΔHR_{7min} (bpm)	-0.04	0.803	0.15	0.269
ΔsBP_{7min} (mmHg)	**-0.41**	**0.002**	**-0.31**	**0.024**
ΔdBP_{7min} (mmHg)	**-0.30**	**0.028**	-0.18	0.188
ΔmBP_{7min} (mmHg)	**-0.39**	**0.004**	**-0.28**	**0.045**
ΔTPR_{7min} (%)	**-0.29**	**0.037**	**-0.33**	**0.017**
Supine HR (bpm)	**0,32**	**0,021**	0,21	0,134
Supine sBP (mmHg),	-0,02	0,878	0,01	0,931
Supine dBP (mmHg)	0,04	0.754	0,08	0,587
Supine mBP (mmHg)	-0,00	0.999	0,01	0,948
Supine CO (1/ml)	-0.35	**0.011**	**-0,36**	0.008
Supine SV (ml)	-0.32	**0.021**	**-0,33**	0.015
Supine TPR (%)	0,25	0.077	**0.27**	**0.048**
$\Delta MAP_{2A\ phase-1}$, (mmHg)	0,14	0.308	0.11	0.437
$\Delta MAP_{2Bphase-2A}$, (mmHg)	-0.14	0.317	-0.20	0.156
$\Delta MAP_{4-baseline}$, (mmHg)	-0.07	0.627	-0.05	0.722
$\Delta HR_{2B-baseline}$, (bpm)	-0.22	0.113	-0.08	0.581
$\Delta HR_{4-baseline,}$ (bpm)	0.11	0.453	0.14	0.304
$\Delta CO_{2Bphase-2A}$ (%)	0.29	0.034	0.32	0.019
$\Delta SV_{2Bphase-baseline,}$ (%)	0.41	0.002	0.34	0,012
$\Delta TPR_{2Bphase-2A}$ (%)	-0.28	0.043	-0.36	0.008

HR, heart rate; sBP, systolic blood pressure; dBP, diastolic blood pressure; mBP, mean blood pressure; SV, stroke volume; CO, cardiac output; TPR, total peripheral vascular resistance; change (Δtilt-supine); CAN MG, presence of cardiovascular autonomic neuropathy; Non-CAN MG, absence of cardiovascular autonomic neuropathy; MG, myasthenia gravis
